# Lung Surfactant Levels are Regulated by Ig-Hepta/GPR116 by Monitoring Surfactant Protein D

**DOI:** 10.1371/journal.pone.0069451

**Published:** 2013-07-29

**Authors:** Taku Fukuzawa, Junji Ishida, Akira Kato, Taro Ichinose, Donna Maretta Ariestanti, Tomoya Takahashi, Kunitoshi Ito, Jumpei Abe, Tomohiro Suzuki, Shigeharu Wakana, Akiyoshi Fukamizu, Nobuhiro Nakamura, Shigehisa Hirose

**Affiliations:** 1 Department of Biological Sciences, Tokyo Institute of Technology, Yokohama, Japan; 2 Life Science Center, Tsukuba Advanced Research Alliance, University of Tsukuba, Tsukuba, Japan; 3 Technology and Development Team for Mouse Phenotype Analysis, RIKEN BioResource Center, Tsukuba, Japan; 4 Faculty of Biomedical Engineering, Toin University of Yokohama, Yokohama, Japan; University of Giessen Lung Center, Germany

## Abstract

Lung surfactant is a complex mixture of lipids and proteins, which is secreted from the alveolar type II epithelial cell and coats the surface of alveoli as a thin layer. It plays a crucial role in the prevention of alveolar collapse through its ability to reduce surface tension. Under normal conditions, surfactant homeostasis is maintained by balancing its release and the uptake by the type II cell for recycling and the internalization by alveolar macrophages for degradation. Little is known about how the surfactant pool is monitored and regulated. Here we show, by an analysis of gene-targeted mice exhibiting massive accumulation of surfactant, that Ig-Hepta/GPR116, an orphan receptor, is expressed on the type II cell and sensing the amount of surfactant by monitoring one of its protein components, surfactant protein D, and its deletion results in a pulmonary alveolar proteinosis and emphysema-like pathology. By a coexpression experiment with Sp-D and the extracellular region of Ig-Hepta/GPR116 followed by immunoprecipitation, we identified Sp-D as the ligand of Ig-Hepta/GPR116. Analyses of surfactant metabolism in *Ig-Hepta^+/+^* and *Ig-Hepta^−/−^* mice by using radioactive tracers indicated that the Ig-Hepta/GPR116 signaling system exerts attenuating effects on (i) balanced synthesis of surfactant lipids and proteins and (ii) surfactant secretion, and (iii) a stimulating effect on recycling (uptake) in response to elevated levels of Sp-D in alveolar space.

## Introduction

Ig-Hepta is a member of the adhesion class of G protein-coupled receptors [1]–[3] and also known as the abbreviated serial name GPR116 [4]. Ig-Hepta is unique in its long extracellular domain comprising immunoglobulin (Ig)-like repeats [5], in its structure composed of three fragments [6], [7], and in high expression predominantly in lung [5]. So far, the physiological function of Ig-Hepta is not known, except a role in adipocytes [8], like many other members of the adhesion class.

The lung alveolar epithelium consists of type I (AT-I) and type II (AT-II) pneumocytes (Fig. S1). AT-I cells are large, thin cells that cover over 90% of the internal surface area of alveolus. AT-I cells are important for gas exchange and alveolar fluid regulation. AT-II cells are cuboidal and located between AT-I cells. AT-II cells contain characteristic lamellar bodies in their cytoplasm and have many known functions, including synthesis and secretion of lung surfactant, fluid transport, and host defense.

Pulmonary surfactant is a complex mixture of lipids and proteins that forms a thin film at the air-liquid interface in the fluid-lined alveoli and prevents alveolar collapse by lowering the surface tension during respiration [9]. The surfactant contains predominantly phospholipids, especially dipalmitoyl-phosphatidylcholine (DPPC), with ∼10% proteins consisting of 4 specific proteins, which can be divided into two groups: (i) the hydrophilic surfactant proteins Sp-A (34–36 kDa) and Sp-D (43 kDa) and (ii) the hydrophobic surfactant proteins Sp-B (8.7 kDa) and Sp-C (4.2 kDa). The low molecular weight Sp-B and Sp-C are intricately associated with the surfactant lipids and regulate the integrity and composition of the surface lipid film, such that it optimally controls interfacial surface tension. Sp-A and Sp-D are surfactant collectins capable of inhibiting foreign pathogens and involved in innate immune responses in the lung [10]. In the case of Sp-D, however, it has been suggested to have dual functional capacity to execute host defense and to regulate pulmonary surfactant homeostasis through the analyses of *Sp-D^−/−^* mice [11]–[15]. The composition and the amount of the heterogeneous lipid-protein mixture that makes up surfactant are tightly regulated but its mechanism is not known [16]. In this paper, we report the functional analysis of Ig-Hepta and identification of its ligand. We generated mice lacking the gene encoding Ig-Hepta. The preliminary result of *Ig-Hepta^−/−^* mice was reported in an abstract form [17], which has recently been confirmed by a loss-of-function approach [18] and a global and conditional gene knockout approach [19]. The mice exhibited a phenotype very similar to that of *Sp-D^−/−^* mice, raising a possibility that the two proteins are functionally correlated in pulmonary surfactant homeostasis.

## Materials and Methods

### Ethics Statement

The animal protocols and procedures were approved by the Institutional Animal Care and Use Committee of Tokyo Institute of Technology.

### Generation of Ig-Hepta^−/−^ Mice and Genotyping

The *Ig-Hepta^−/−^* mouse line was generated essentially as previously described [20]–[23], and housed and used according to the NIH Guide for the Care and Use of Laboratory Animals. For detailed methods, see Supplementary Material and Methods.

### Extraction of Bronchoalveolar Lavage Fluid (BALF)

BALF was obtained according to Ikegami *et al.* [14]. Mice were injected intraperitoneally with pentobarbital (Abbott Laboratories, Chicago, USA) to achieve deep anesthesia. The distal aorta was cut to exsanguinate each animal. A 26-gauge blunt needle was inserted into the trachea with the use of a 1.0-ml syringe attached to the needle, and the lungs were filled with 0.7-ml aliquots of phosphate-buffered saline (PBS). The fluid was then withdrawn by syringe three times for each aliquot. Alveolar lavage was performed twice and the samples were pooled (∼1 ml). The samples were supplemented with protease inhibitors (10 µM leupeptin, 1 µM pepstatin, 5 µg/ml aprotinin and 1 mM phenylmethylsulfonyl fluoride) and cleared by centrifugation at 1,000×*g* for 10 min at 4°C. The resulting supernatants were further centrifuged at 20,000×*g* for 30 min at 4°C.

### Measurement of Saturated Phosphatidylcholine (SatPC) Pool Size and Protein Contents

BALF of *Ig-Hepta* ^+/+^ and *Ig-Hepta* ^−*/*−^ mice (*n* = 4 for each genotype) were obtained as described above. Remaining whole lung tissues were homogenized in 5 ml saline (0.9% NaCl). SatPC was purified and quantified from aliquots of both BALF and lung tissue homogenates according to the method of Ikegami et al. [24], [25]. The amount of protein was determined using the rest of the samples by BCA Protein Assay Kit (Pierce, Rockford, USA).

### Precursor Incorporation Assay

*Ig-Hepta* ^+/+^ and *Ig-Hepta* ^−*/*−^ mice (*n* = 4 for each condition) were given a intraperitoneal injections of 8 µl saline/body weight (g) containing 0.5 µCi [^3^H]choline/body weight (g) (GE Healthcare, Piscataway, USA) according to the method of Ikegami et al. [24], [25]. At 8 and 48 hr after the [^3^H]choline injection, BALF was recovered with 5 ml saline and the remaining lung tissue was homogenized in 5 ml saline at 4°C. SatPC was isolated from whole BALF and lung tissue homogenates as described above, quantified, and measured for radioactivity.

### Immunoprecipitation

Cells were extracted with 200 µl of 1% Triton X-100 in PBS containing protease inhibitors by sonication. The cell extracts were cleared by centrifugation at 16,000×*g* for 30 min at 4°C. To analyze the interaction of secreted SP-D-Myc with secreted FLAG-tagged Ig-Hepta deletion proteins, culture medium containing SP-D-Myc (500 µl) was mixed with the same volume of culture medium containing FLAG-tagged Ig-Hepta deletion constructs. The cell lysates and the culture medium were then incubated with 20 µl of anti-FLAG M2 affinity beads overnight at 10°C. After washing three times with PBS, the beads were eluted with 20 µl of Laemmli buffer.

### Statistical Analysis

Data are expressed as means ± SEM. Statistical comparisons were performed with Student’s *t* test. *P*<0.05 was considered statistically significant.

## Results

### Generation of *Ig-Hepta^−/−^* Mice

**Figure 1:**
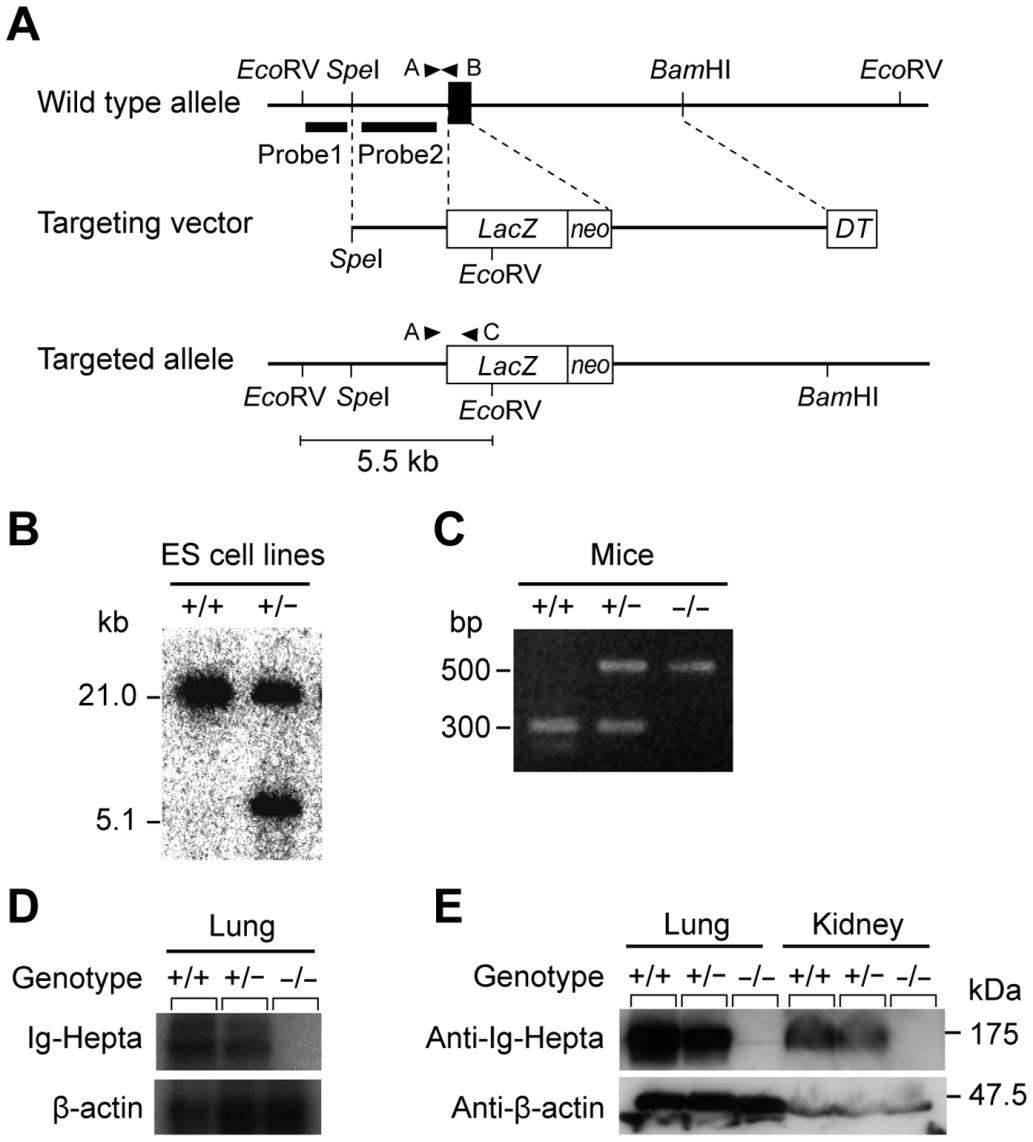
Targeted disruption of the mouse *Ig-Hepta/GPR116* gene. A, Schematic maps of the *Ig-Hepta* locus, the targeting vector, and the recombinant locus after targeting. Dark box denotes the exon containing the initiation codon. Dotted lines delineate the regions of homology between the wild-type allele and the vector. The nuclear localization signal-β-galactosidase gene (*LacZ*), the neomycin phosphotransferase gene (*neo*), and the diphtheria toxin-A gene (*DT*) are shown as an open box. The positions of the probes used for Southern blot analysis (closed bars) and primers for PCR analysis (closed triangles) are also shown. B, Southern blot analysis of ES cells. Genomic DNAs isolated from wild-type (+/+) and mutant (+/−) ES cell clones were digested with EcoRV and blotted. Fragments obtained from wild-type allele (21.0 kb) and targeted allele (5.5 kb) were detected by external probe (probe 1) and internal probe (probe 2). The data is representative of blotting with probe 1. C, Determination of mice genotype by PCR analysis of tail-derived DNA. The 500-bp fragment amplified with primers A and B shows the presence of the wild-type allele (+/+); the 300-bp fragment amplified with primers A and C indicates the mutant allele (−/−). Both alleles are detected in heterozygous mice (+/−). D, Northern blot analysis of Ig-Hepta-deficient mice. RNA samples from lung of wild-type (+/+), heterozygous (+/−) and homozygous (−/−) mice were analyzed with probes for mouse Ig-Hepta and β-actin mRNAs. E, Western blot analysis of Ig-Hepta-deficient mice. Protein samples from lung (40 µg) and kidney (100 µg) were analyzed by Western blotting with anti-Ig-Hepta polyclonal antibody (upper panel) and anti-β-actin monoclonal antibody (Sigma) (lower panel). Molecular mass markers are indicated on the right.

The *Ig-Hepta* gene was disrupted in embryonic stem cells using standard homologous recombination techniques (Fig. 1A). The second exon containing the initiation codon of Ig-Hepta was replaced with the *lacZ* gene and neomycin resistance gene. Homologous recombination was confirmed by Southern blot analysis with 5′-external and internal probes (Fig. 1B). Fifteen of the 238 colonies that survived positive/negative selection contained the mutant allele. Two targeted clones were injected into ICR 8-cell embryos to generate chimeric mice, and one clone gave rise to germ line transmission by backcross mating with C57BL/6J mice. The heterozygous mice were crossed to produce homozygous mice that were identified by PCR analysis (Fig. 1C). Of the 135 offspring analyzed, 35 (26%) were wild type and 35 (26%) were homozygous for the disrupted allele, consistent with the expected Mendelian ratio. Homozygous mutant mice had normal growth and development.

Our previous study has shown that Ig-Hepta is highly expressed in lung and to a much lesser extent in kidney and heart [5]. Northern blot analysis of lung mRNA showed that *Ig-Hepta* transcripts were completely absent in homozygous mutant mice, but detected in wild-type and heterozygous mice (Fig. 1D). The lack of expression of Ig-Hepta in homozygous null mutant mice was also confirmed at the protein level by Western blot analysis (Fig. 1E).

### Analysis of Ig-Hepta Expression

Since the targeting construct, used for disruption of the *Ig-Hepta* gene, is designed to generate *Ig-Hepta*-null/*lacZ*-knockin mice that express the ß-galactosidase reporter gene tagged with the nuclear localization signal (*nls-lacZ*) under the control of the *Ig-Hepta* promoter, it is possible to determine the Ig-Hepta-expressing cells by detecting the *nls-lacZ* product (namely β-galactosidase accumulated in the nucleus).

**Figure 2:**
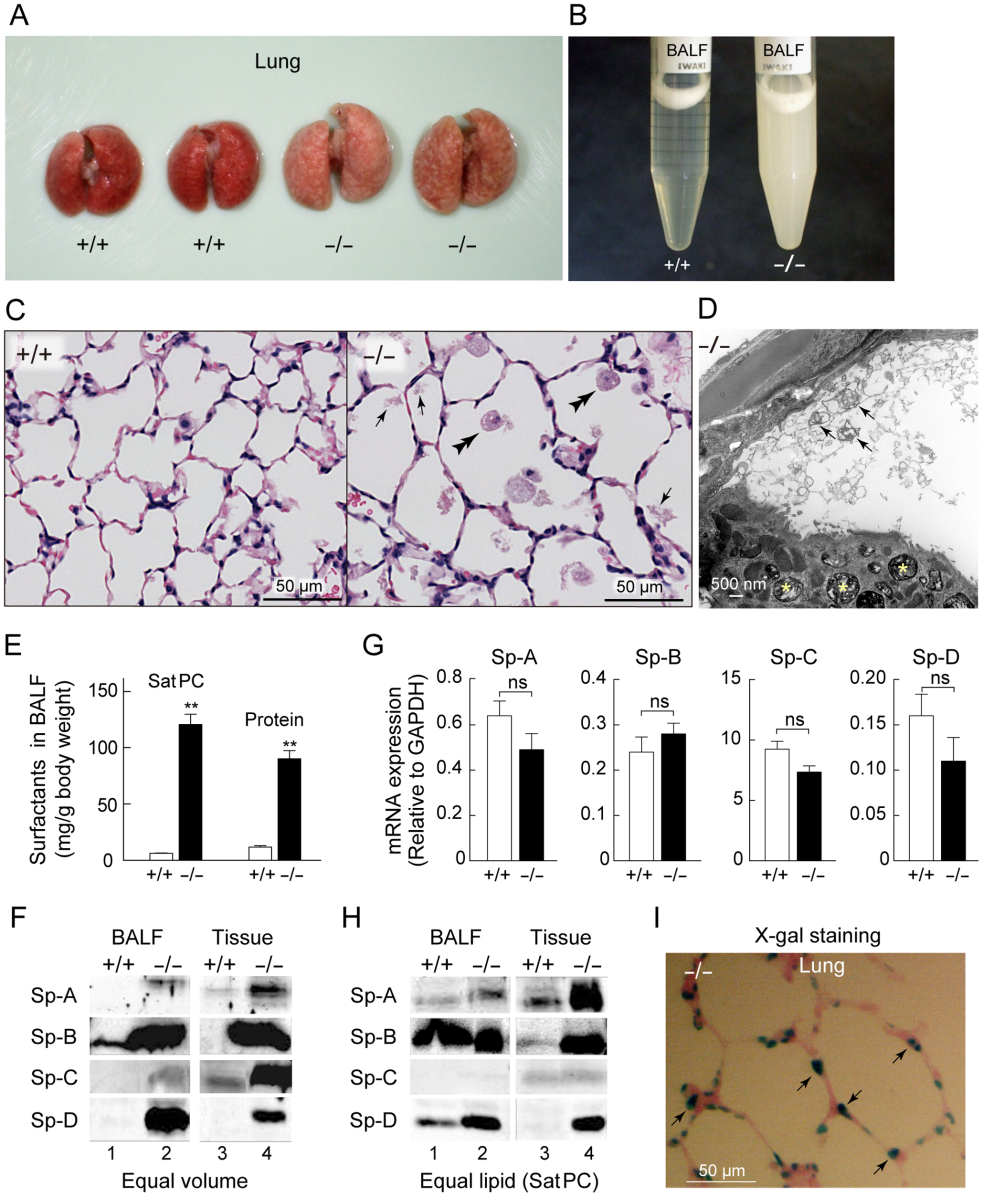
Accumulation of surfactants in the lung of *Ig-Hepta^−/−^* mice. A, Lungs of *Ig-Hepta^+/+^* and *Ig-Hepta^−/−^* mice. B, A picture of BALF from *Ig-Hepta^+/+^* and *Ig-Hepta^−/−^* mice. C, Hematoxylin-eosin staining of lung sections of *Ig-Hepta^+/+^* and *Ig-Hepta^−/−^* mice. Arrows point to surfactant aggregates and double arrowheads indicate macrophages whose number was increased in the lung of *Ig-Hepta^−/−^* mice. The average diameter of the cross section of alveoli was 43±4 µm for *Ig-Hepta^+/+^* mice and 63±8 µm for *Ig-Hepta^−/−^* mice (*P* = 0.03). D, Transmission electron microscopy of the lung of an *Ig-Hepta^−/−^* mouse. Arrows and asterisks indicate tubular myelin in the alveolar lumen and lamellar bodies in an AT-II cell, respectively. E, Quantification of surfactant lipid (SatPC) and total proteins in BALF from *Ig-Hepta^+/+^* and *Ig-Hepta^−/−^* mice. **P<0.001. F, Western blot analyses of surfactant proteins. Equal volumes of BALFs or tissue homogenates were loaded. G, Quantification of mRNA expressions of surfactant proteins in the lung of *Ig-Hepta^+/+^* and *Ig-Hepta^−/−^* mice. Average ratios compared to GAPDH mRNA were as follows (mean ± SEM, *n* = 4). Sp-A: 0.64±0.06 (+/+), 0.49±0.07 (−/−), *P* = 0.16; Sp-B: 0.24±0.03 (+/+), 0.28±0.02 (−/−), *P* = 0.33; Sp-C: 9.2±0.7 (+/+), 7.3±0.5 (−/−), *P* = 0.06; and Sp-D: 0.16±0.02 (+/+), 0.11±0.03 (−/−), *P* = 0.28. ns, not significant. H, Western blot analyses of surfactant proteins. BALFs or tissue homogenates containing equal amounts of SatPC were loaded. In the protein basis, lanes 1–4 contain 10, 4, 40, and 7 µg proteins, respectively. I, Activity staining of β-galactosidase with its substrate X-gal for determining Ig-Hepta-expressing cells (green) in the mouse lung.

**Figure 3:**
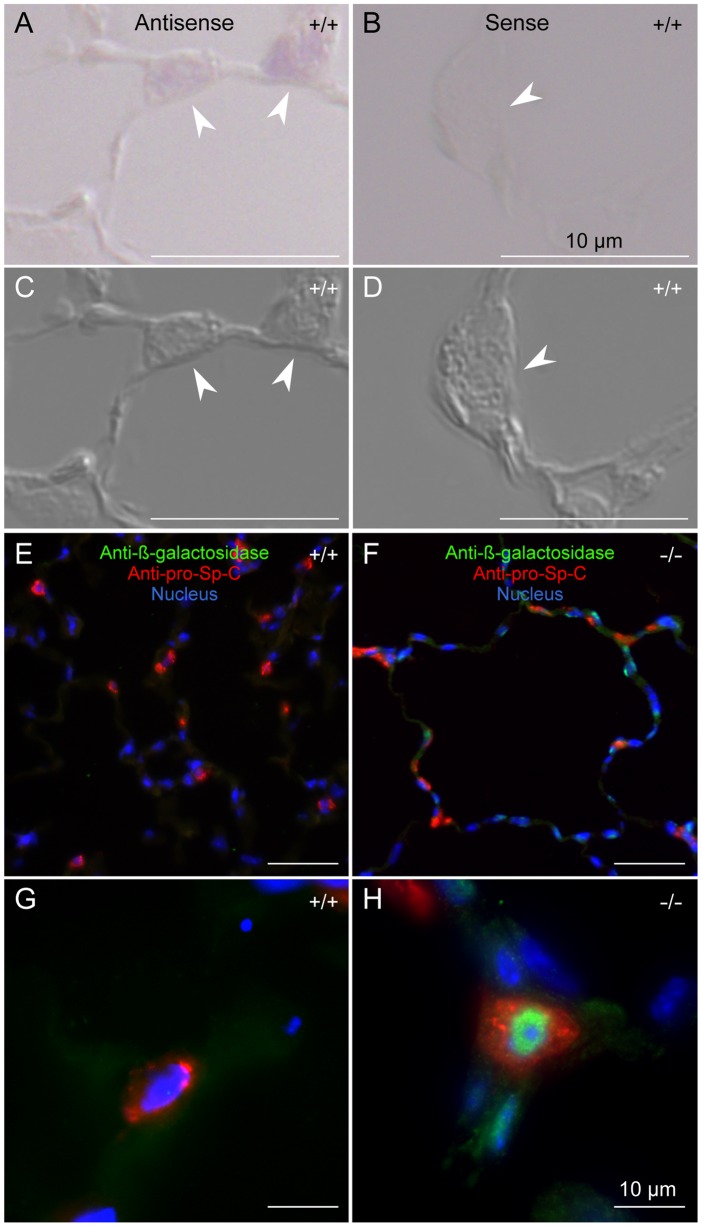
Ig-Hepta-expressing cells determined by in situ hybridization and immunohistochemical double-staining. A and B, In situ hybridization with antisense and sense probes to *Ig-Hepta* mRNA. C and D, Differential interference contrast images of (A) and (B), respectively. Arrowheads indicate the cells that exhibit the appearance of type II cell. Ig-Hepta signal is detected in these cells. E and F, Double-staining pictures of *Ig-Hepta^+/+^* and *Ig-Hepta^−/−^* mouse lung sections. G and H, Higher magnification images of type II cell. FITC fluorescence (green) represents β-galactosidase signal, which indicates altered *Ig-Hepta* expression in *Ig-Hepta^−/−^* mice. Cy3 fluorescence (red) shows pro-Sp-C, an AT-II cell marker. Nuclei were stained by Hoechst 33342 (blue). Scale bars, 10 µm.

**Figure 4:**
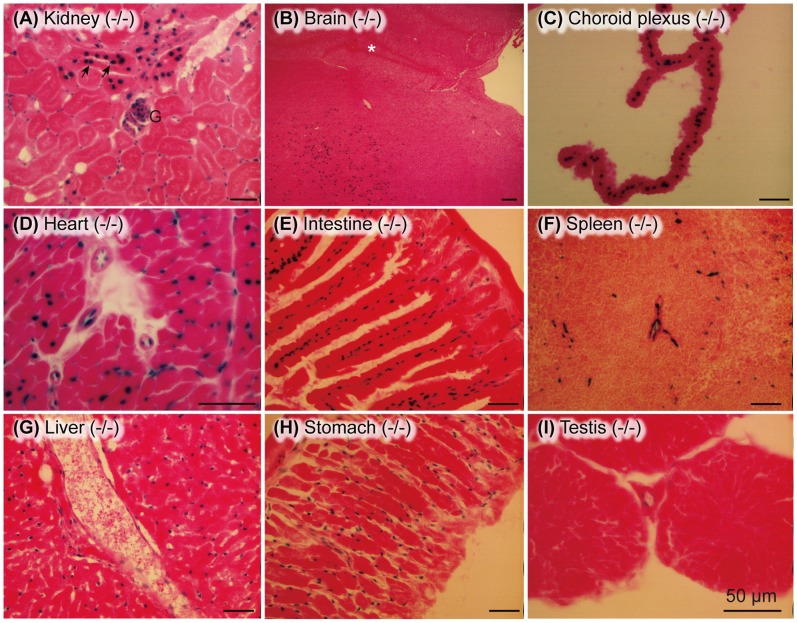
Ig-Hepta/GPR116-expressing cells determined by β-galactosidase assay. Expression of Ig-Hepta was determined in various tissues, other than the lung (Fig. 2I), including renal cortex (A), post thalamic region of the brain (B), choroid plexus (C), heart (D), intestine (E), spleen (F), liver (G), stomach (H), and testis (I). In the kidney, intercalated cells, glomerular endothelial cells, and capillary endothelial cells around the renal tubules were stained. Concerning the other tissues examined, most capillary endothelial cells were stained. Arrows in (A) indicate intercalated cells of renal collecting duct. G, glomerulus. Asterisk in (B) shows hippocampus. Scale bars, 50 µm.

Analysis of *nls-lacZ* expression by β-galactosidase histochemistry revealed a strong expression in AT-II cells in the lung (Fig. 2I, blue), which was confirmed by in situ hybridization and immunohistochemistry (Fig. 3). A moderate expression was seen in the intercalated cells of the kidney (Fig. 4A) and a weak but significant staining was observed in the capillary endothelial cells of various tissues (Fig. 4B–I).

### Abnormalities in Lung Morphology and Surfactant Levels in Ig-Hepta^−/−^ Mice

Under non-perfused conditions, mottled appearance and hypertrophy of the lung were evident at autopsy of *Ig-Hepta* ^−/−^ mice (Fig. 2A). The lung weight was ∼1.3 times higher in the *Ig-Hepta* ^−/−^ mice (0.38±0.02 g for *Ig-Hepta^+/+^*, 0.37±0.02 g for *Ig-Hepta^+/−^*, and 0.50±0.03 g for *Ig-Hepta^−/−^* mice, *n* = 8–12, *P* = 0.003 for +/+ vs −/−).

Fig. 2B shows relative amounts of surfactants present in the alveolar space, which were recovered as bronchoalveolar lavage fluids (BALF). The BALF from *Ig-Hepta* ^−/−^ mice contained markedly elevated levels of surfactants. The materials accumulated in the alveolar space of *Ig-Hepta* ^−/−^ mice were also seen on tissue sections; extracellular surfactant aggregates were rarely seen in wild-type lungs but frequently observed in the lungs of *Ig-Hepta* ^−/−^ mice (Fig. 2C, arrows). The materials were better defined by electron microscopy (Fig. 2D), some of which had a tubular structure (Fig. 2D, arrows) that is very similar to tubular myelin known to be composed of surfactants and transported to plasma membrane by lamellar bodies (Fig. 2D, asterisks). Quantitative analyses of the BALF indicated a 12-fold increase in saturated dipalmitoyl-phosphatidylcholine (DPPC, a lipid characteristic of alveolar surfactant) and a 7-fold increase in surfactant proteins (Fig. 2E).

Western blot analyses of BALF revealed that levels of the surfactant proteins Sp-A, Sp-B, Sp-C, and Sp-D were all highly elevated in *Ig-Hepta* ^−/−^ mice (Fig. 2F, left column). Although the amounts of surfactants contained in BALF were markedly different between *Ig-Hepta* ^+/+^ and *Ig-Hepta* ^−/−^ mice, the surfactant composition (namely the ratio of surfactant proteins and SatPC contained in BALF) was almost identical (Fig. 2H, left column). Surfactant protein levels in the lung tissue were also greatly elevated in *Ig-Hepta* ^−/−^ mice (Fig. 2F, right column). Excess surfactant proteins seem to be synthesized compared to their partner lipids (Fig. 2H, right column).

**Figure 5:**
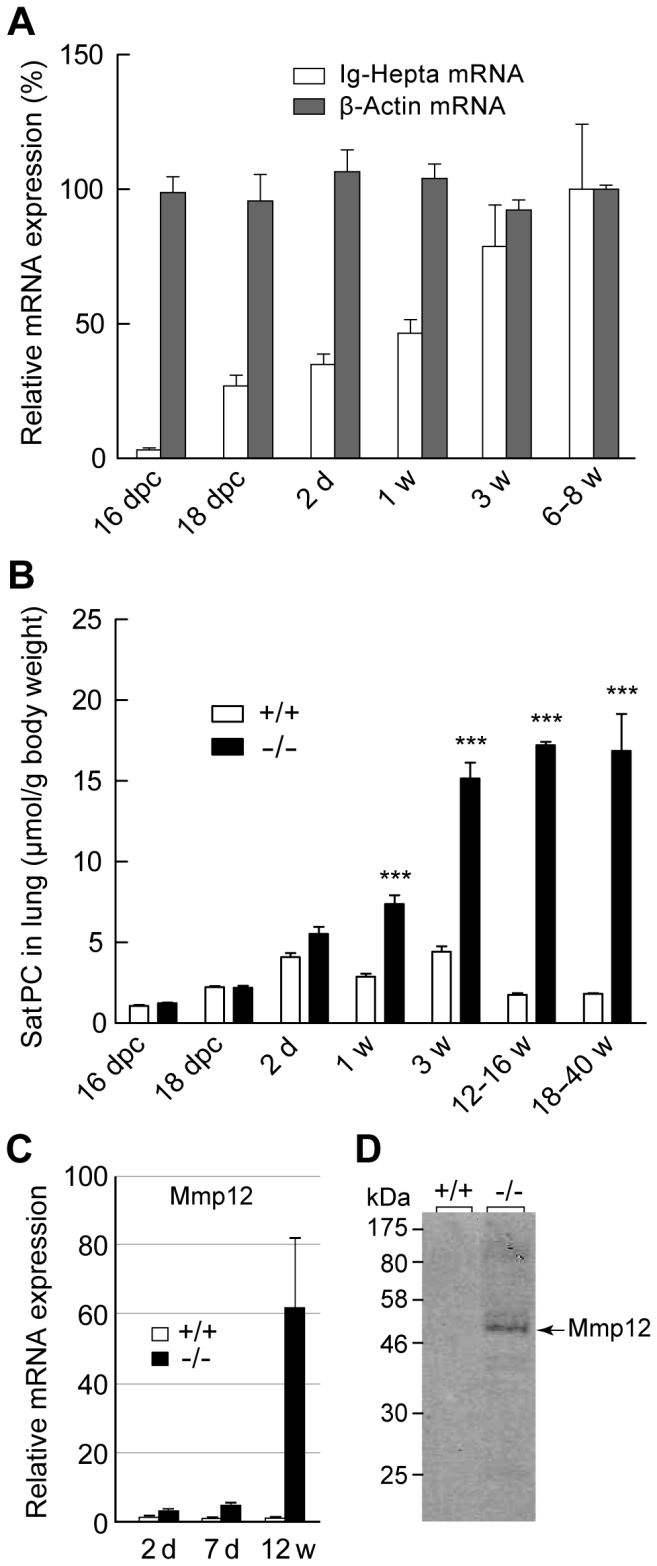
Age-dependent expression of *Ig-Hepta* mRNA and accumulation of surfactants and Mmp12. A, Expression of *Ig-Hepta* mRNA was quantified by real-time PCR using mRNA preparations from fetal, neonatal, young, and adult lungs of *Ig-Hepta^+/+^* mice. dpc, days post coitum. B, Age-dependent accumulation of surfactant lipid (SatPC) in the lung of *Ig-Hepta^+/+^* and *Ig-Hepta^−/−^* mice (*n* = 3, ***P<0.001). C and D, Induction of expression of Mmp12 mRNA and its protein product in the lung of *Ig-Hepta^−/−^* mice revealed by real time PCR and Western blotting, respectively.

*Ig-Hepta* ^−/−^ mice spontaneously developed a pulmonary emphysema-like symptom characterized by a reduced number and an increased volume of alveoli (Fig. 2C), which was associated with a marked increase of matrix metalloproteinase 12 (Mmp12; Fig. 5C, D). A similar emphysema-like symptom has been reported in *Sp-D* ^−/−^ mice; namely, targeted disruption of the *Sp-D* gene has been shown to cause the accumulation of surfactants, increase in the number of lipid-laden foamy macrophages, and emphysema, which was associated with increased production of matrix metalloproteinases [15], [26]. Moderate hypertrophy of alveolar type II cells was observed in *Ig-Hepta* ^−/−^ mice (Fig. 3G, H). The average size of AT-II cells was 10.2±0.3 µm for *Ig-Hepta^+/+^* mice and 11.5±0.2 µm for *Ig-Hepta* ^−/−^ mice (*P* = 0.02).

### Regulation of Synthesis of Surfactant Proteins at the Translational Level

Based on the above observation that the amounts of surfactant proteins are markedly increased in the lung of *Ig-Hepta* ^−/−^ mice, we simply expected that the mRNA levels are also upregulated. A real-time PCR (qPCR) analysis, however, indicated that this is not the case; no significant changes were observed in the message levels of the surfactant proteins between the *Ig-Hepta* ^+/+^ and *Ig-Hepta* ^−/−^ mice (Fig. 2G).

### Increased Number and Enlargement of Alveolar Macrophages in *Ig-Hepta^−/−^* Mice

**Figure 6:**
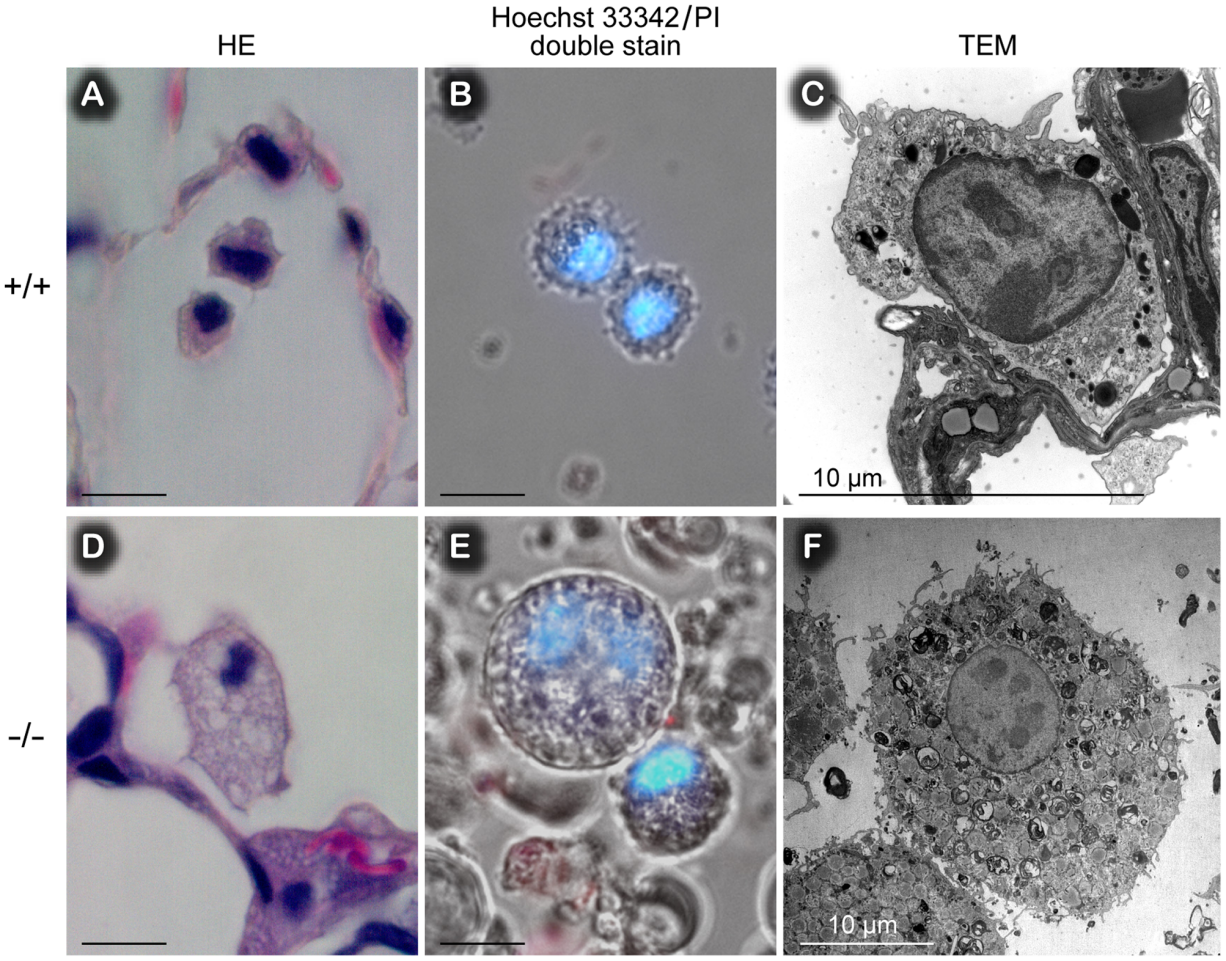
Hypertrophic alveolar macrophages in *Ig-Hepta^−/−^* mice (D–F) compared to *Ig-Hepta^+/+^* mice (A–C). A and D, Hematoxylin and eosin stains. B and E, Viability of macrophages confirmed by a Hoechst/propidium iodide double stain apoptosis detection kit. C and F, Transmission electron microscope images of an alveolar macrophage of *Ig-Hepta^+/+^* and *Ig-Hepta^−/−^* mice. Scale bars, 10 µm.

Giemsa staining of cells recovered in BALF revealed a progressive increase of the number of alveolar macrophages in *Ig-Hepta^−/−^* mice (Fig. S2); the increase became evident around 3 weeks of age, reached a peak at 8–12 weeks, and remained increased thereafter. A marked morphological alteration was also observed in alveolar macrophages. Most alveolar macrophages of *Ig-Hepta* ^−/−^ mice were abnormally large and contained abundant cytoplasmic vesicles (Fig. 2C, double arrowheads; Fig. 6D–F), suggesting excessive accumulation of surfactants. In alveolar macrophages, no significant expression of *Ig-Hepta* mRNA was observed by RT-PCR using mRNA preparations from *Ig-Hepta^+/+^* mouse lung, suggesting that Ig-Hepta is not expressed, or if any, at very low levels in alveolar macrophages (Fig. S3). This result is consistent with the very recent report by Yang et al. [19] that macrophage-specific deletion of the *Gpr116* (*Ig-Hepta*) gene caused no significant alteration in surfactant levels, and suggests that the foamy appearance is not a direct effect of *Ig-Hepta* deletion and rather a secondary effect of the surfactant accumulation. These results imply that the lack of Ig-Hepta results in uncontrolled secretion of surfactants from the alveolar type II cells and accumulation of surfactants in alveoli.

### Increased Synthesis and Secretion of DPPC in *Ig-Hepta^−/−^* Mice

Macrophages seem to be functioning normally and taking up surfactants as much as possible, as evidenced by their large and foamy structure (Fig. 6D–F), but the amount of the surfactants secreted exceeds the phagocytic ability of macrophages. To address this issue, we next analyzed the metabolism of surfactants in the *Ig-Hepta* ^−/−^ mice.

**Figure 7:**
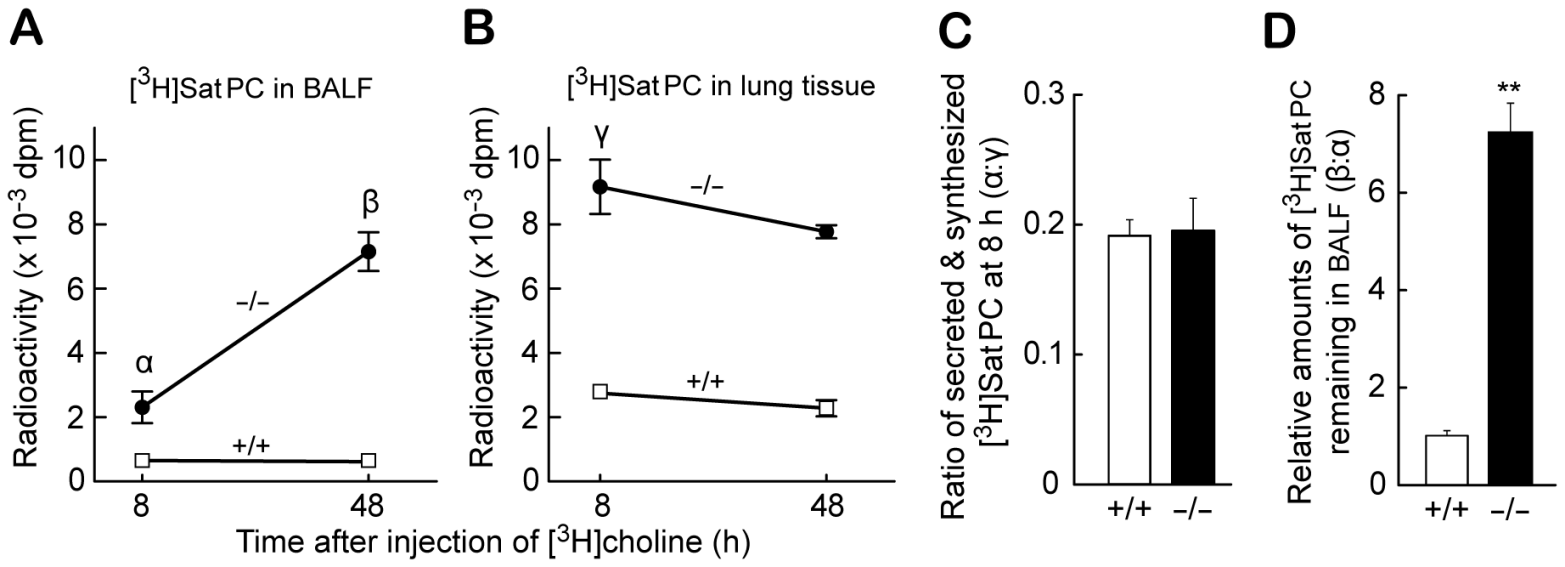
Increased synthesis and reduced catabolism of DPPC in *Ig-Hepta^−/−^* mouse lung monitored by radiotracer uptake. A and B, Radioactivity of SatPC in BALF and lung tissues corrected by their lung weights at 8 and 48 h. [^3^H]Choline uptake was measured at 8 h to evaluate surfactant synthesis and secretion, and at 48 h to assess surfactant catabolism (*n* = 4 for each condition). C, Amounts of SatPC secreted relative to those synthesized, which were evaluated by the ratio of [^3^H]choline incorporation into SatPC in BALF at 8 h [marked “α” in (A)] to that in lung tissues at 8 h [marked “γ” in (B)]. D, Reduced SatPC catabolism in *Ig-Hepta^−/−^* mice evaluated by the ratio of [^3^H]choline incorporation into SatPC in BALF at 48 h [marked “β” in (A)] to that in BALF at 8 h [marked “α” in (A)]. Values are means ± SE. *P<0.05, **P<0.001.

Incorporation of [^3^H]choline into DPPC is generally used for monitoring the biosynthesis of surfactant lipids since (i) the concentration of DPPC is uniquely high in the pulmonary surfactant and (ii) the disaturated nature of DPPC enables its separation from the ordinary phospholipids present in the body by osmium tetroxide column chromatography. To measure the rate of synthesis of DPPC, a major component of pulmonary surfactant, [^3^H]choline was administered intraperitoneally to the *Ig-Hepta* ^+/+^ and *Ig-Hepta* ^−/−^ mice and its incorporation into DPPC was quantified by the osmium tetroxide chromatography of total lipids extracted from lung tissues and BALF at 8 h (for measuring synthesis and secretion) and 48 h (for measuring reuptake). The [^3^H]choline incorporation assay indicated that the surfactant synthesis and secretion by type II cells are increased (Fig. 7A, B) and the surfactant uptake by the type II cell is reduced in the *Ig-Hepta* ^−/−^ mice (Fig. 7D).

### Ontogeny of Ig-Hepta mRNA Expression and Accumulation of Surfactants

To know the timing of Ig-Hepta expression in the lung, the expression of Ig-Hepta mRNA was quantified in fetal, neonatal, young, and adult lungs of wild-type mice by real-time PCR (Fig. 5A). *Ig-Hepta* mRNA expression became significant at 18 days post coitum (dpc), increased until 3 weeks old, and reached its maximum levels at 3–6 weeks old. This onset of the expression roughly coincides with maturation of the surfactant system in AT-II cells.

Fig. 5B shows SatPC contents in the lungs of *Ig-Hepta* ^+/+^ and *Ig-Hepta* ^−/−^ mice before and after birth up until the age of 40 weeks. By 2 days old, the SatPC levels were not significantly different between the *Ig-Hepta* ^+/+^ and *Ig-Hepta* ^−/−^ mice. At the age of 1 week, however, the difference became significant and at the age of 12–16 weeks, approximately 10 times higher levels were observed in *Ig-Hepta* ^−/−^ mice (*n* = 4–5, *P*<0.01).

### Identification of Sp-D as the Ligand for Ig-Hepta

The phenotypes described above for *Ig-Hepta^−/−^* mice are very similar to those reported for *Sp-D^−/−^* mice, including (i) accumulation of a large amount of surfactants, (ii) enlarged alveoli, (iii) hypertrophy of AT-II cells, (iv) decreased surfactant uptake by AT-II cells, (v) accumulation of enlarged foamy macrophages, and (vi) enhanced expression of Mmp12 [11], [12], [27]. The similarities imply a close or direct relationship between Sp-D and Ig-Hepta. Sp-D has recently been demonstrated to have a domain that recognizes an immunoglobulin motif [28]. We therefore decided to explore the possibility of the ligand-receptor relationship of Sp-D and Ig-Hepta by monitoring their interaction by immunoprecipitation.

**Figure 8:**
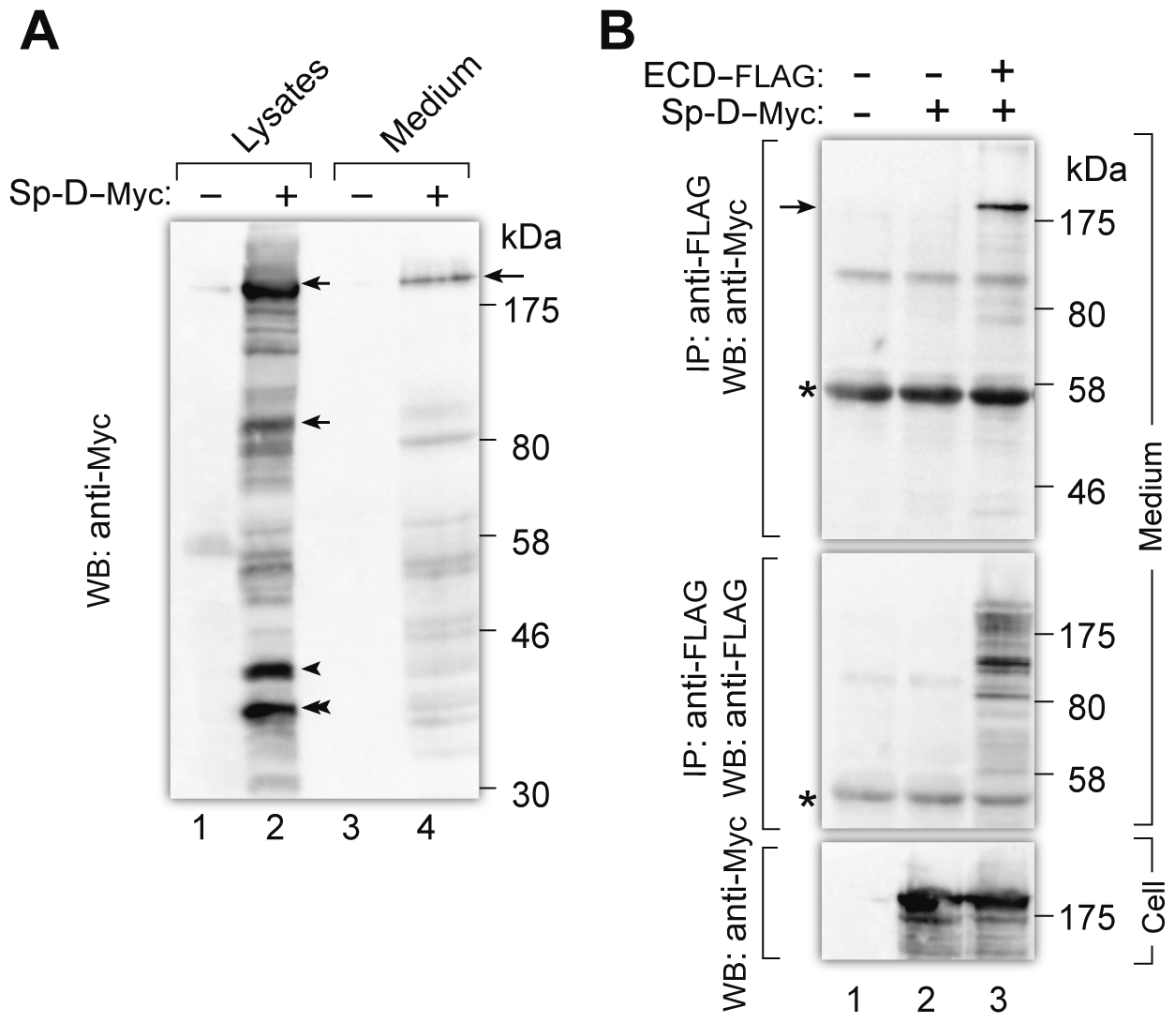
Interaction of Sp-D-Myc with the N-terminal extracellular domain (ECD) of mouse Ig-Hepta. A, Confirmation of expression of Sp-D-Myc in 293T cells and its secretion into culture medium. 293T cells were transiently transfected with mock (lanes 1 and 3) or Sp-D-Myc (Lanes 2 and 4). At 48 h after transfection, culture medium was harvested and the cells were extracted with 1% Triton in PBS. The cell lysates (20 µg of proteins, lanes 1 and 2) and the medium (20 µl, lanes 3 and 4) were analyzed by Western blotting (WB) with anti-Myc antibody. Arrows and arrowhead indicate multimeric forms and a monomer of Sp-D-Myc, respectively. Double arrowhead indicates non-glycosylated Sp-D-Myc [42]. B, 293T cells were transfected with mock (lane 1) or Sp-D-Myc alone (lane 2) or along with ECD-FLAG (lane 3). At 48 h after transfection, culture media were subjected to immunoprecipitation (IP) with anti-FLAG M2 beads followed by Western blotting (WB) with anti-Myc antibody (top panel). The blot was reprobed with anti-FLAG antibody (middle panel). The cell lysates (20 µg of proteins) used for the immunoprecipitation were analyzed by Western blotting with anti-Myc antibody (bottom panel). Asterisks indicate the bands corresponding to IgG.

We constructed a mammalian expression vector encoding a C-terminally Myc-tagged mouse Sp-D (Sp-D-Myc). Sp-D-Myc was transfected into 293T cells, and the cell lysates and culture medium were analyzed by Western blotting with anti-Myc antibody. As shown in Fig. 8A, Sp-D-Myc was detected as high molecular weight oligomers in both the lysates and medium, indicating that Sp-D-Myc was secreted as multimers; Sp-D monomer has a molecular mass of ∼43 kDa and is known to be secreted as a dodecamer formed by assembly of four trimeric subunits [29]. To see the interaction of Sp-D and the extracellular domain (ECD) of Ig-Hepta, Sp-D-Myc was transfected into 293T cells along with ECD-FLAG, and 48 h after transfection, culture medium was subjected to immunoprecipitation with anti-FLAG antibody. Western blotting of the immunoprecipitates revealed co-precipitation of Sp-D-Myc and ECD-FLAG (Fig. 8B). The result that Sp-D specifically binds to the extracellular region of Ig-Hepta suggests that Sp-D is a strong candidate for an endogenous Ig-Hepta ligand.

## Discussion

In the present study, in an attempt to clarify the physiological roles of Ig-Hepta, we generated an *Ig-Hepta^−/−^* mouse line using a targeting vector containing the *lacZ* gene and found that Ig-Hepta is relatively highly expressed in the pulmonary AT-II cells. These locations led us to suspect its link with maintaining the surfactant homeostasis, and therefore to determine and compare the amounts of surfactants in bronchoalveolar lavage fluids of *Ig-Hepta^+/+^* and *Ig-Hepta^−/−^* mice. The result provided us an important clue suggesting that Ig-Hepta is involved in the control of surfactant levels.

**Figure 9:**
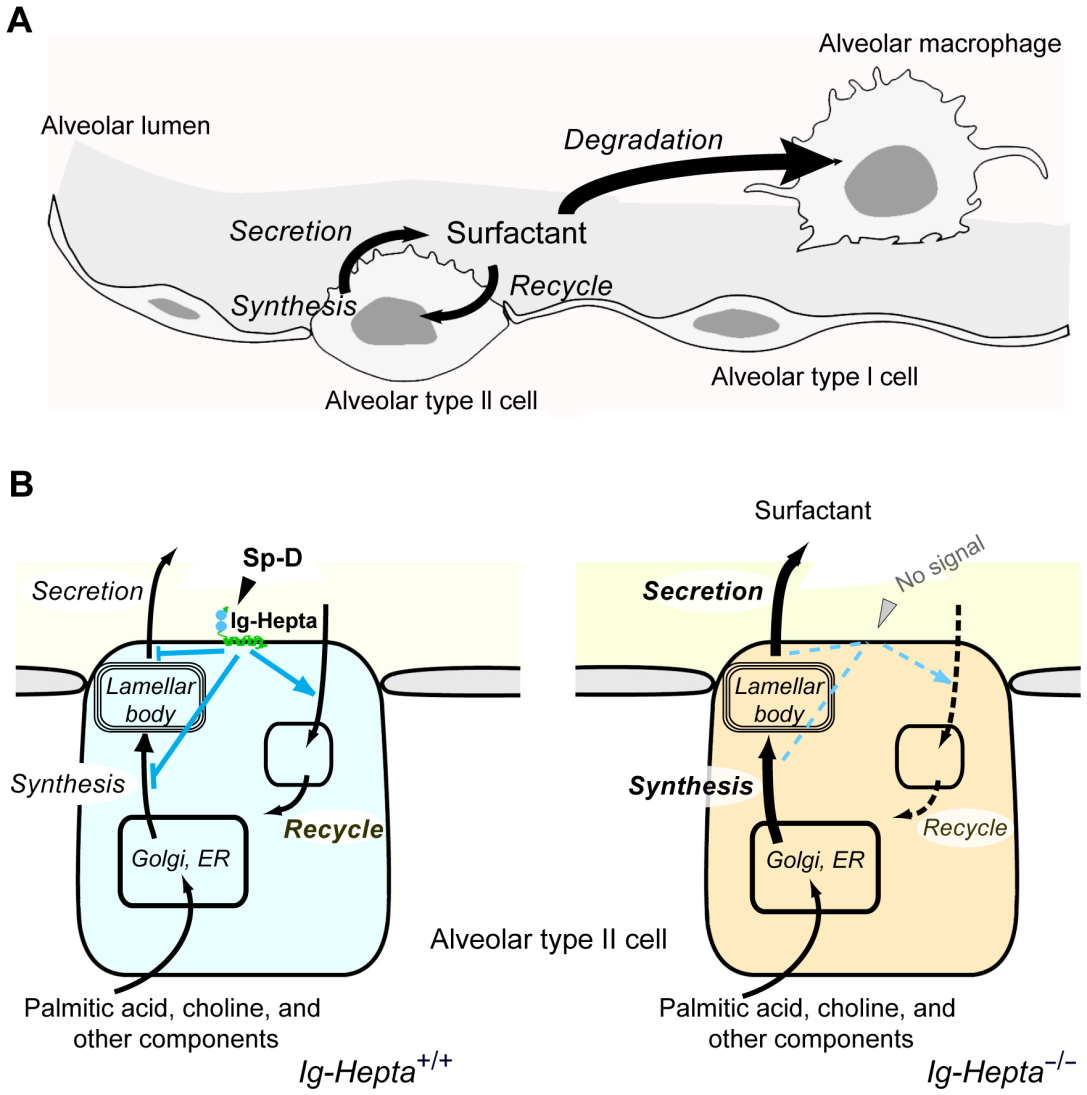
Schematic illustration of roles of Ig-Hepta in maintaining pulmonary surfactant homeostasis. A, Metabolic and catabolic pathways of pulmonary surfactants. B, Surfactant homeostasis in lungs of *Ig-Hepta* ^+/+^ and *Ig-Hepta* ^−/−^ mice. According to our working model, the Ig-Hepta/GPR116 signaling system exerts attenuating effects on (i) balanced synthesis of saturated phosphatidylcholine (SatPC) and surfactant proteins and (ii) surfactant secretion, and (iii) a stimulating effect on recycling (uptake) by monitoring the levels of Sp-D in alveolar space. Deletion of Ig-Hepta/GPR116 results in massive accumulation of surfactants in alveolar space, which in turn activates phagocytosis of macrophages as evidenced by appearance of enlarged foamy macrophages. The activated macrophages gradually release matrix metalloproteinase 12 (Mmp12) and inflammatory cytokines & chemokines, which attract white blood cells (an inflammatory response as a secondary effect; unpublished observation; Supplementary Fig. S1). Concerning the regulation of biosynthesis of the surfactant proteins, a translational regulation seems to be a key mechanism for coordinated production of surfactant lipids and proteins since no significant changes were seen in the mRNA levels of the surfactant proteins between *Ig-Hepta* ^+/+^ and *Ig-Hepta* ^−/−^ mice.

Our finding may have important implication on respiratory physiology. Currently, the molecular mechanism for controlling and maintaining proper surfactant levels is still not completely understood. It is generally accepted that pulmonary surfactants are internalized by phagocytosis of macrophages and endocytosis of AT-II cells [27], [30]–[32] for degrading or recycling. Failure in surfactant uptake and degradation by macrophages may result in accumulation of surfactants over the alveolar epithelial surface. However, this is not the case in *Ig-Hepta^−/−^* mice because (i) the number of macrophages is not reduced in the knockout mice, rather it is increased, and (ii) their phagocytic activity is not likely to be regulated by the Ig-Hepta signaling system since Ig-Hepta is not expressed on macrophages. The most likely scenario is therefore that Ig-Hepta is involved in the control of secretion of surfactants by AT-II cells and, in *Ig-Hepta^−/−^* mice, there is a defect somewhere in the processes of uptake and recycling and/or secretion of de novo synthesized surfactants (Fig. 9).

Despite the markedly increased levels of surfactant proteins, their message levels were not elevated significantly in *Ig-Hepta^−/−^* mice. This unexpected discrepancy can be explained by either decreased turnover by lack of surfactant protein breakdown or increased protein synthesis regulated at the translational level. Although we did not determine the rate of protein breakdown in *Ig-Hepta^−/−^* mice, the fact that surfactant protein levels are markedly elevated within AT-II cells of *Ig-Hepta^−/−^* mice may suggest that the amounts of surfactant proteins are mainly regulated at the level of translation. This mode of regulation would be advantageous in optimizing the rate of surfactant protein synthesis in order to tune it with the synthesis of surfactant lipids because pulmonary surfactants are a lipid-protein mixture whose composition must be kept fairly constant. This coordinated synthesis seems to be disrupted in *Ig-Hepta* ^−/−^ mice, and excess surfactant proteins are synthesized compared to their partner lipids owing to the lack of feedback inhibition. It may worth mentioning that translational regulation has been established in the synthesis of hemoglobin, which requires balanced synthesis of globins and heme (reviewed by Chen [33]).

By a coexpression experiment with Sp-D and the extracellular region of Ig-Hepta followed by immunoprecipitation, we identified Sp-D as a strong candidate for the ligand of Ig-Hepta. This ligand–receptor relationship fits well with the fact that Sp-D is also present in plasma [34], which is expected to serve as the ligand for Ig-Hepta in capillary endothelial cells of various tissues. The receptors for Sp-D have been identified in macrophages, which are regulators of inflammation and innate immunity, including the calreticulin/CD91 complex, SIRPα, CD14 and toll-like receptors [35]–[37]. Recently, Fournier et al. [28] have demonstrated that Sp-D binds to the membrane-proximal Ig domain of SIRPα. This Ig-domain recognition property of Sp-D may also indirectly support the ligand-receptor relationship of Sp-D and Ig-Hepta that has two Ig-like extracellular domains. Given the number of immune and surfactant-related functions of Sp-D, it is not surprising that multiple candidate receptors have been described for Sp-D.

Previous studies have established the role of granulocyte-macrophage colony-stimulating factor (GM-CSF) in regulating surfactant catabolism in alveolar macrophages [38]–[40] (Fig. S1). However, GM-CSF does not regulate the surfactant uptake by either macrophages for degradation or AT-II cells for recycling [41]. In contrast to the GM-CSF system, the Ig-Hepta signaling system seems to regulate, by sensing extracellular levels of Sp-D, the intracellular events in AT-II cells including (i) coordination of synthesis of surfactant proteins and surfactant lipids, (ii) secretion of surfactants into the alveolar lining, and (iii) recycling of surfactants. These considerations indicate that the Sp-D–Ig-Hepta signaling system plays a central role in surfactant homeostasis.

Very recently, Bridges et al. [18] have generated a transgenic mouse line with a targeted mutation of the *Gpr116* locus (*Gpr116^Δexon17^*), which produces a protein product devoid of the transmembrane domain encoded by exon 17. *Gpr116^Δexon17^* mice exhibited a phenotype very similar to that reported here including a profound accumulation of surfactant, which was associated with increased SatPC synthesis and the presence of enlarged lipid-laden alveolar macrophages. In *Gpr116^Δexon17^* mice, as in *Ig-Hepta/Gpr116^−/−^* mice reported here, no significant alterations were observed in the mRNA level of the surfactant proteins. The only difference between our study and that of Bridges et al. is that we observed marked increases in the levels of both surfactant proteins and lipids while Bridges et al. reported only moderate increases (less than 3-fold) of Sp-B and Sp-C in BALF and a significant decrease of Sp-A and an increase of Sp-D in tissue homogenates. The difference may be due to the accumulation of Gpr116^Δexon17^, a truncated form of Gpr116, within AT-II cells and serve as a clue to clarifying the regulatory mechanism of surfactant proteins. Also very recently, Yang et al. [19] have reported production of global and conditional knockout (KO) mice lacking the *Gpr116* gene totally or in a cell type-specific manner, and demonstrated that Gpr116 on the AT-II cell plays an indispensable role in lung surfactant homeostasis by regulating reuptake of surfactants into AT-II cells. The phenotypes are very similar to ours except the observation of Yang et al. that no significant increase in radiolabeled SatPC in the KO alveolar space following intravenous injection of [^14^C]choline into *Gpr116* WT or KO mice. The reason for this discrepancy is not clear and should be clarified by future studies.

## Supporting Information

Figure S1 **Schematic illustration of pulmonary surfactant homeostasis.**
(TIF)

Figure S2 **Increased number of alveolar macrophage in** ***Ig-Hepta^−/−^*** **mice.**
(TIF)

Figure S3 **Undetectable levels of** ***Ig-Hepta*** **mRNA in macrophages.**
(TIF)

Materials and Methods S1 (DOCX)
