# Supplementary figures and images for: Lung Surfactant Levels are Regulated by Ig-Hepta/GPR116 by Monitoring Surfactant Protein D
Source: PLoS One. 2013 Jul 29;8(7):e69451. doi: 10.1371/journal.pone.0069451 (PMC3726689; doi:10.1371/journal.pone.0069451)

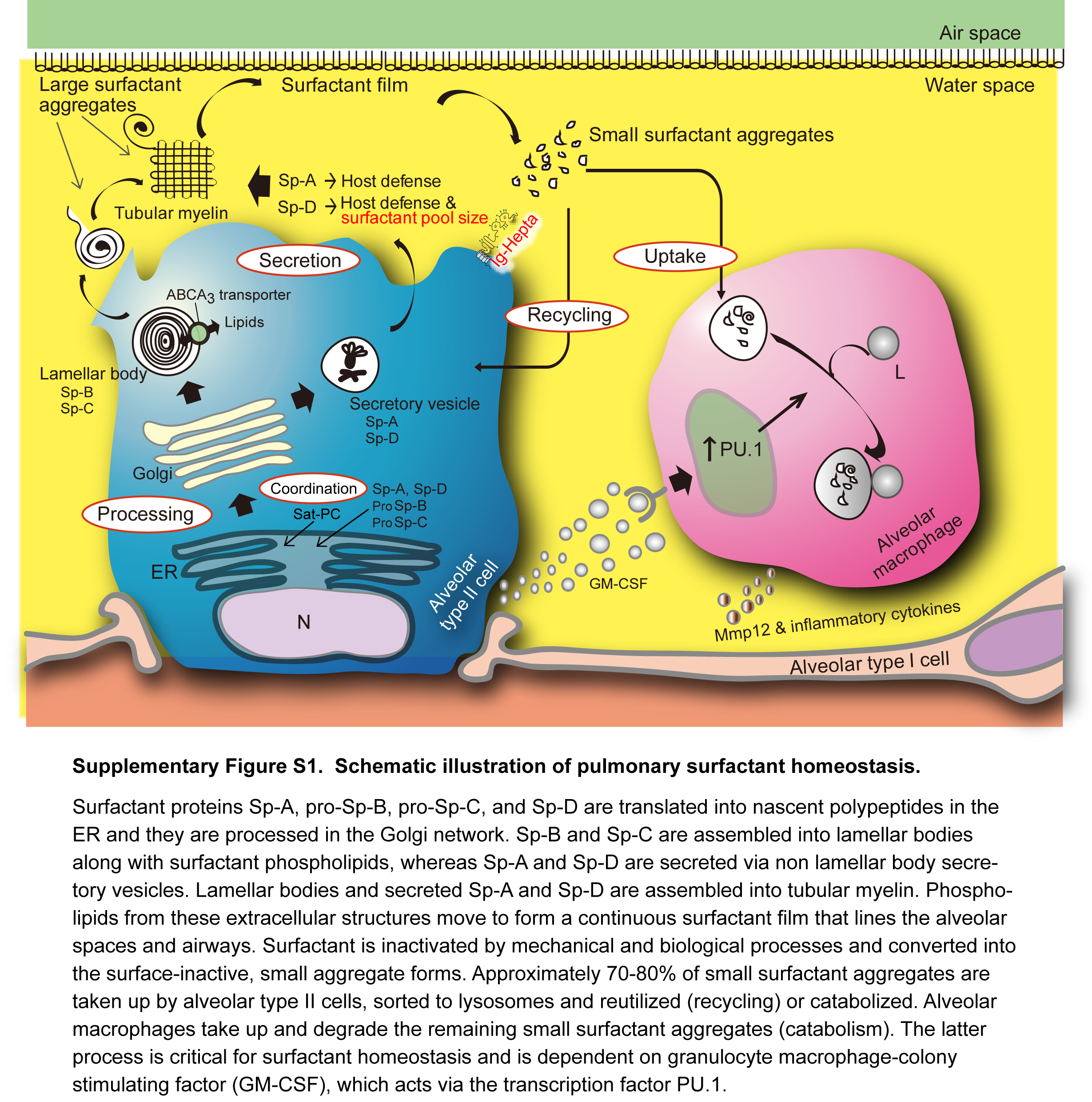

Supplement: Figure S1 — Schematic illustration of pulmonary surfactant homeostasis. (TIF) [file pone.0069451.s001.tif]

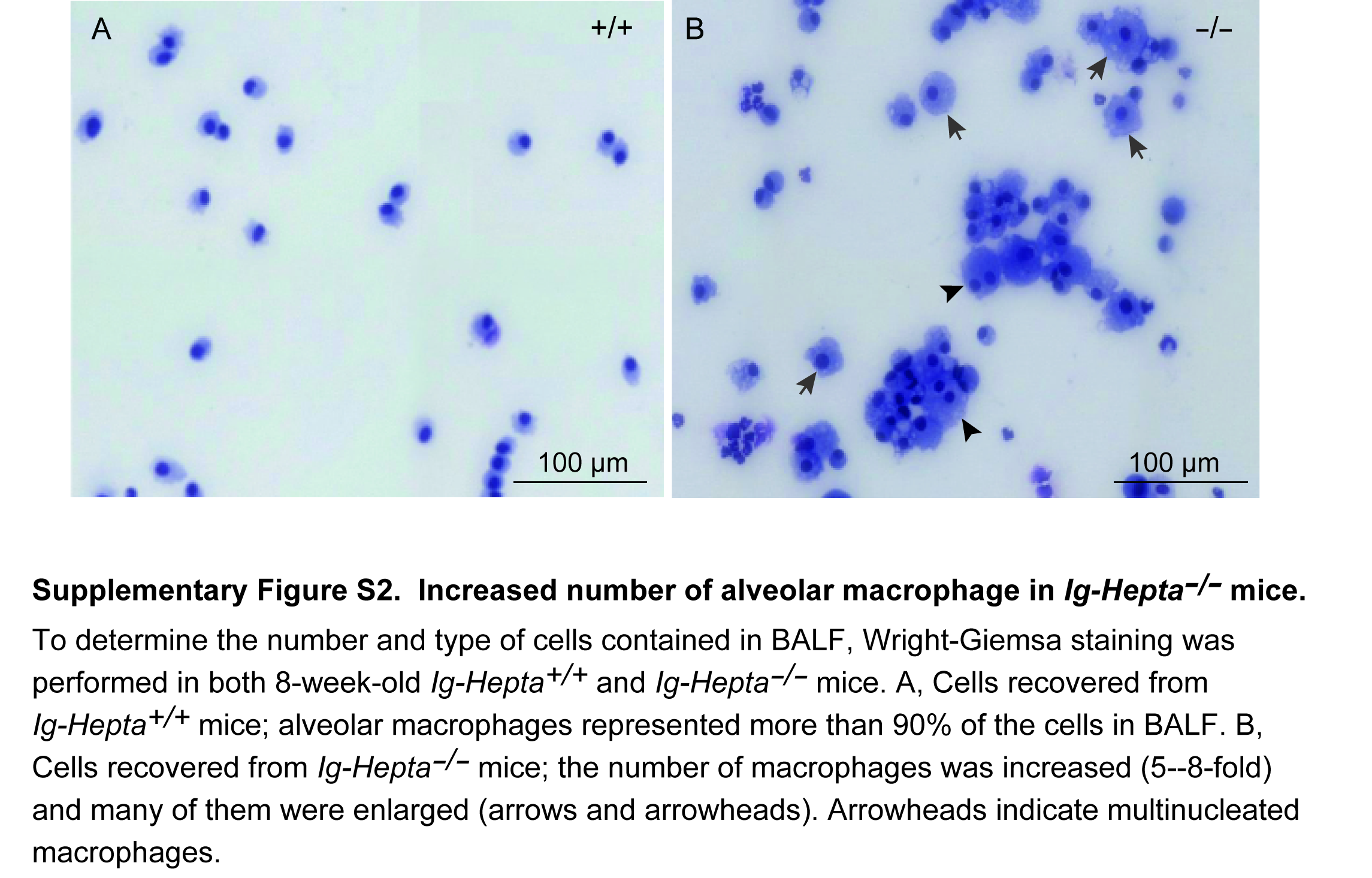

Supplement: Figure S2 — Increased number of alveolar macrophage in Ig-Hepta−/− mice. (TIF) [file pone.0069451.s002.tif]

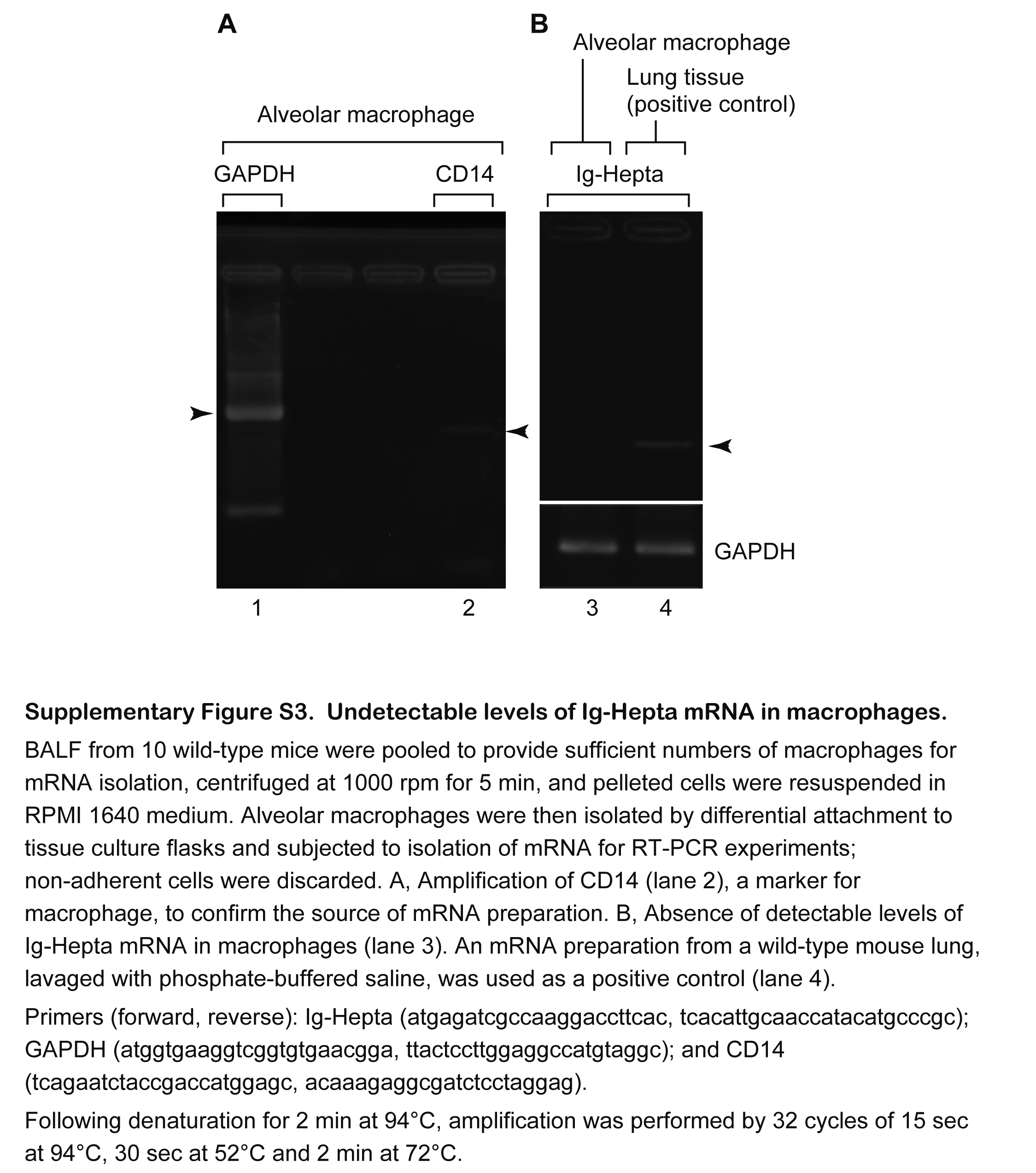

Supplement: Figure S3 — Undetectable levels of Ig-Hepta mRNA in macrophages. (TIF) [file pone.0069451.s003.tif]
