# Supplementary material for: Lung Surfactant Levels are Regulated by Ig-Hepta/GPR116 by Monitoring Surfactant Protein D
Source: PLoS One. 2013 Jul 29;8(7):e69451. doi: 10.1371/journal.pone.0069451 (PMC3726689; doi:10.1371/journal.pone.0069451)
Supplement: Materials and Methods S1 — (DOCX) [file pone.0069451.s004.docx]

**Supplementary Materials and Methods**

Materials

Anti-Sp-A (AB3420), Sp-B (AB3780), and Sp-D (AB3434) polyclonal antibodies were purchased from Chemicon International, CA, USA; Anti-Sp-C (sc-13979) and anti-CD14 (sc-9150) antibodies were from Santa Cruz Biotechnology, CA, USA; Anti-Aquaporin 5 antibody was from Calbiochem, CA, USA; Restriction enzymes were purchased from Takara, Kyoto, Japan; ECL-plus reagent was obtained from Amersham Pharmacia Biotech, Uppsala, Sweden; Immobilon polyvinylidene difluoride membrane was purchased from Millipore, Tokyo, Japan; Isogen was from Nippon Gene, Tokyo, Japan; Hybond N^+^ nylon membranes and Ready-To-Go DNA labeling kit were from GE Healthcare, Piscataway, NJ, USA; 5-bromo-4-chloro-3-indolyl β-d-galactopyranoside (X-gal), potassium ferricyanide, and potassium ferrocyanide were from Wako Pure Chemical Industries, Osaka, Japan; 3-aminopropyltriethoxysilane (APS)-coated glass slide was from Matsunami, Osaka, Japan; Mount-quick was from Daido Sangyo Co, Saitama, Japan; BCA protein assay kit was from Pierce, IL, USA.

Generation of *Ig-Hepta^–/–^* mice and genotyping

The genomic DNA containing the *Ig-Hepta* locus was isolated from a phage library from C57BL/6 mice [1]. To construct a targeting vector, the *Ig-Hepta* initiation codon and its neighboring sequence (AGATGA) was replaced with the *Nco*I recognition site (CCATGG) by PCR. The 260-bp fragment of the mouse *Ig-Hepta* gene between *Nco*I site and *Spe*I site was replaced with the nuclear localization signal-lacZ cassette and neomycin resistance gene cassette derived from pMC1neoPolyA (Stratagene). The amino acid sequence of nuclear localization signal located at 5’ end of *LacZ* was as follow: MASGRNSAKRMKEAKEKRQEQIAKRRRLSSLRASLA. The TT2 ES cells were grown on embryonic fibroblast feeder cells according to the procedure of Yagi et al. [2]. ES cells were transfected by electroporation with the linearized targeting vector and screened by negative/positive selection with the diphtheria toxin-A and G418, respectively [3]. Homologous recombination was detected by Southern blotting of *Eco*RV-digested genomic DNA using 5’-external and 5’-internal probes. Chimeric mice were generated by injecting the ES cells into ICR 8-cell embryos [4]. Chimeric offspring were bred with C57BL/6J female mice. F1 offspring were screened for germ-line transmission of the mutant allele by Southern blot analysis and PCR analysis with primers A and B to detect the wild-type allele; A and C to detect the null allele. The primers used for identification of genotype were as follows: A, 5’-TTCTCCCCATCCTCTGGGACATGTTTGCCT-3’; B, 5’-AAGGTTGAACAATGGGCTCTGTTGGTGTGC-3’; C, 5’-CTCAGGAAGATCGCACTCCAGCCAGCTTTC-3’.

Ig-Hepta knockout strain was crossed back 10 times to C57BL/6J mice. Ig-Hepta homozygous knockout mice (*Ig-Hepta*^−/−^) were initially obtained by Ig-Hepta heterozygous brother-sister inbreeding and were sustained by homozygous brother- sister inbreeding afterward. In all experiments, C57BL/6J wildtype mice of the almost same age were used as experimental control.

Bronchoalveolar lavage fluid (BALF) lipid and protein measurements

Mice were anesthetized with Nembutal (Abbott Laboratories) by intraperitoneal injection. The abdominal cavity was opened, and the diaphragm was cut. The 20-gauge plastic cannula (SR-0T2032C, Terumo) was inserted into the trachea. With the use of 2.5-ml syringe fitted on the cannula, the lungs were lavaged 4 times with 1 ml of phosphate-buffered saline. Bronchoalveolar lavage fluid (BALF) was subfractionated into a pellet fraction and two surfactant fractions [5]. Cell pellet was isolated by centrifugation at 150 × *g* for 10 min at 4 °C. The supernatant was centrifuged at 20,000 × *g* for 60 min at 4 °C. This resulted in a subfractionation into large aggregate (LA) surfactant in the pellet and small aggregate (SA) surfactant in the supernatant. The protein concentration of each fraction was determined with BCA protein assay kit according to the manufacture’s protocol. The total phospholipid contents in the cell-free BALF were measured using enzymatic method by Koto-Biken Medical Laboratories (Tokyo, Japan).

Western blot analysis of tissue homogenates

Tissues collected from mice were homogenized with 5 volumes of 1% Triton X-100 containing protease inhibitors at 4 °C overnight. The detergent-soluble fractions were mixed with 5× Laemmli buffer (300 mM Tris containing 10% SDS, 40% glycerol, and 0.025% bromphenol blue, pH 6.8) in the presence of 5% β-mercaptoethanol, unless specifically mentioned. Proteins were electrophoresed through SDS-PAGE and then transferred to polyvinylidene difluoride (PVDF) membrane. The membrane was blocked with 5% nonfat dry milk, 0.05% Tween 20 in Tris-buffered saline (TBS, 150 mM NaCl, 10 mM Tris-HCl, pH 8) (T-TBS) for 1 h at room temperature. The membrane was washed 3 times for 10 min each with T-TBS and subsequently treated overnight at 4 °C with the primary antibody in T-TBS. The production of anti-Ig-Hepta antibody was described previously [8]. After washing the blots 3 times for 10 min each with T-TBS, the membrane was incubated for 1 h at 25 °C with a horseradish peroxidase-conjugated antibody diluted 1:30000 in T-TBS. The blots were washed 3 times for 10 min each with T-TBS. The membrane was then developed using the ECL-Plus detection system.

Western blot analysis of surfactant proteins

BALFs of *Ig-Hepta^+/+^* and *Ig-Hepta^-/-^* mice were obtained as described in Experimental Procedures. Remaining whole lung tissues were homogenized in 5-ml saline (0.9% NaCl). Each sample was homogenized with protein inhibitors and 5% of Triton X-100. Prepared samples were mixed with 5 × Laemmli buffer in the presence of 5% β-mercaptoethanol (for anti-Sp-A and anti-Sp-D) and without the presence of β-mercaptoethanol (for anti-Sp-B). The samples were then separated on 12% SDS-PAGE gel (for anti-Sp-A and anti-Sp-D) and 17% SDS-PAGE gel (for anti-Sp-B) and then electrophoretically transferred to Immobilon-FL transfer membrane (Millipore). The membrane was blocked with 5% skim milk in 0.05% Tween 20 in Tris-buffered saline (TBS, 150 mM NaCl, 10 mM Tris-HCl, pH 8) (TBS-Tween) for 1 h at room temperature. The membrane was washed 3 times for 10 min each with TBS-Tween and subsequently probed overnight at 4 °C with the primary antibody in TBS-Tween. After washing, blots were incubated with secondary antibody conjugated to Alexa Fluor 680 (1:10,000 dilution) in TBS-Tween for 1 h at room temperature. Immunoblots were washed with TBS-Tween 3 times for 10 min, washed with TBS-Tween containing 0.01% SDS once for 5 min, rinsed in TBS-Tween for another 10 min, and then visualized using the Odyssey infrared imaging system (LI-COR Bioscience, Lincoln, USA).

Detection of β-galactosidase activity

Tissues were collected from mice at 8 weeks of age. Frozen tissue sections were transferred to APS-coated glass slides, dried, fixed (0.2% w/v glutaraldehyde in 5 mM EGTA, 2 mM MgCl_2_, 100 mM Na_2_HPO_4_, pH 7.3, 10 min), rinsed (2 mM MgCl_2_, 0.01% sodium deoxycholate, 0.01% Nonidet-P40, 100 mM Na_2_HPO_4_, pH 7.3), and stained (rinse buffer containing 1 mg/ml 5-bromo-4-chloro-3-indolyl β-d-galactopyranoside (X-gal), 5 mM potassium ferricyanide, 5 mM potassium ferrocyanide) at 37 °C for 2 h. After staining, the slides were rinsed with phosphate-buffered saline, washed for 10 min with deionized water, counterstained with 1% eosin for 30 sec, dehydrated, and mounted with Mount-quick. The X-gal stock solution (40 mg/ml) was prepared by dissolving X-gal with dimethyl sulfoxide and stored at −20°C.

Immunohistochemistry

Ig-Hepta^+/+^ and Ig-Hepta ^−/−^ mice were anesthetized by intraperitoneal injection of pentobarbital, and the distal aorta was cut to exsanguinate the animal. The trachea was exposed and a 20-gauge blunt needle was inserted into the trachea and ligated. After instillation with 4% (wt/vol) paraformaldehyde in 0.1 M phosphate buffer, pH 7.4, from a height of 26 cm for 5 min, the lungs were dissected out of the chest cavity, minced, and immersed with the same cold fixative for 2 h. Tissues were then washed in phosphate-buffered saline (PBS: 137 mM NaCl, 2.7 mM KCl, 6.5 mM Na_2_HPO_4_, 1.5 mM KH_2_PO_4_, pH 7.4) containing 10% sucrose at 4°C. Sucrose density was consecutively changed to 16, 18, and 20%. The lungs were then washed with PBS containing 20% sucrose followed by PBS containing 50% Tissue-Tek OCT compound (Sakura Finetek, Tokyo, Japan) and frozen in Tissue-Tek OCT compound at −80°C.

Frozen sections were prepared by cryostat (6 µm thick) at −25°C, mounted on Matsunami Adhesive Silane (MAS)-coated glass slides (Matsunami, Osaka, Japan), and air-dried for 30 min. The sections were washed in PBS, permeabilized with 0.2% Triton X-100 in PBS for 10 min, and blocked with 5% fetal bovine serum (FBS) in PBS for 1 h at room temperature. The samples were then incubated with goat anti-β-galactosidase antiserum (1:1,000 dilution, Promega) and anti-human pro-surfactant protein C (Pro Sp-C) rabbit serum (1:1,000 dilution, Chemicon, Temecula, USA) in PBS containing 5% FBS for 16 h at 4°C. Afterward, the samples were incubated with secondary antibodies conjugated to Alexa Fluor 488 or 594 (1:2,000 dilution, Invitrogen, Carlsbad, USA) and Hoechst 33342 (0.1 µg/ml, Nacalai, Kyoto, Japan) in PBS containing 5% FBS for 1 h at room temperature. Fluorescence was detected using a fluorescence microscope equipped with a digital charge-coupled device (CCD) camera (AxioCam HRm, Carl Zeiss, Oberkochen, Germany) and processed with AxioVision 4.1 software (Carl Zeiss).

In situ hybridization

A 773-bp DNA fragment corresponding to the nucleotide positions 635–1407 of mouse Ig-Hepta (Gpr116, Genbank accession number NM_001081178) was subcloned into pGEMT-Easy vector (Promega, Madison, USA) and was used for generation of sense or antisense RNA probes. The lung of Ig-Hepta^+/+^ mice were inflation-fixed at 26-cm H_2_O pressure with 4% paraformaldehyde (PFA) in 0.1 M phosphate buffer (pH 7.4) for 5 min, excised, post-fixed in the same fixative for ~24 h at 4°C, and then prepared for paraffin sections (6 μm thick). For in situ hybridization, the sections were hybridized with digoxigenin labeled RNA probes at 60°C for 16 h. The bound label was detected using nitro blue tetrazolium and 5-bromo-4-chloro-3-indolyl phosphate (NBT-BCIP). The sections were counterstained with Kernechrot (Muto Pure Chemical, Tokyo, Japan). The images were obtained with a microscope equipped with a CCD camera (AxioCam HRc).

Northern blot analysis

Total RNA from the lungs of 12-week-old mice was isolated with Isogen. Ten micrograms of total RNA were electrophoresed in 1% formaldehyde-agarose gels and then transferred to Hybond N^+^ nylon membranes by capillary blotting using 20 × SSC as the transfer buffer. After transfer, the membranes were baked for 2 h at 80 °C and prehybridized for 1 h at 68 °C in PerfectHyb hybridization solution (Toyobo). The probes were labeled with [α-^32^P]dCTP (3,000 Ci/mmol, 110 TBq/mmol) using Ready-To-Go DNA labeling kit. The membranes were then hybridized with ^32^P-labeled probe in the same buffer at 68 °C for 16 h. After hybridization, the membranes were washed twice with 1 × SSC and 0.1% SDS for 1 h at 60 °C. The membranes were exposed to imaging plates (Fuji Film, Tokyo, Japan) in a cassette for 1 day. The results were analyzed using a Fuji BAS2000 Bio-image analyzer (Fuji Film).

Electron microscopy

Lungs from anesthetized wild-type mice and homozygous mutant mice (12 weeks old) were collected and fixed with 4% (w/v) paraformaldehyde and 2.5% glutaraldehyde. The tissues were processed by standard procedures including tannic acid staining. Ultrathin sections were cut and examined with an electron microscope (Model H-7500, Hitachi, Tokyo, Japan).

RNA isolation and quantitative real-time PCR

Lung RNAs were isolated from *Ig-Hepta*^+/+^ or *Ig-Hepta*^−/−^ mice by performing acid guanidinium thiocyanate-phenol-chloroform extraction with Isogen (Nippon Gene, Tokyo, Japan). Tissues were homogenized in Isogen (5 ml Isogen/mouse), followed by chloroform extraction, isopropanol precipitation, and 75% (vol/vol) ethanol washing of precipitated RNA. The obtained RNA preparations were dissolved in diethyl pyrocarbonate-treated water, and their concentrations were measured spectrophotometrically at 260 nm. Samples were obtained from five *Ig-Hepta*^+/+^ and five *Ig-Hepta* ^−/−^ mice.

Single-stranded cDNA was prepared from 5 μg total RNA with oligo(dT) primer in a 20-μl reaction volume using Superscript III First-Strand System (Invitrogen) and finally diluted in 140 μl diethyl pyrocarbonate-treated water. Real time PCR was set up using 1.0 μl cDNA, 12.5 μl 2 × SYBR Green Master Mix (Takara Bio Inc, Shiga, Japna), and 50 nM each of forward and reverse primers in a total volume of 25 μl. The real-time PCR was run for 45 cycles on the Thermal Cycler Dice Real Time System (Takara) according to the manufacturer’s protocol. The primer sets used for quantification were as follows: Sp-A (NM_023134), 5’-GGAGCTTCAGACTGCACTCTAC-3’ and 5’-CCACTGACAGCATGGATCCT-3’; Sp-B (NM_147779), 5’-GTCGCCAAGTGCTTGATGTCTAC-3’ and 5’-CAGGCCTACATGATTGCAGATGG-3’; Sp-C (NM_011359), 5’-GCTTTCGCTAGAAAACTCCAGAAC-3’ and 5’-ACAGGGTGCTCACAGCAAG-3’; Sp-D (NM_009160), 5’-AAAGGAAAACTACAGCGTCTAGAGG-3’ and 5’-TCCAACACTTCGGCCATCAG-3’; Ig-Hepta (NM_001081178), 5’-CCCGTAGTGAAAAACAGAGA-3’ and 5’-AGCAGGAAGTAGAACGTGAA-3’; Mmp12 (NM_008605), 5’-CCTGGTATTCAAGGAGATGCAC-3’ and 5’-GGGGTCCATTATTGACTTTGG-3’; and glyceraldehyde 3-phosphate dehydrogenase (GAPDH, NM_008084), 5’-ACCACAGTCCATGCCATCAC-3’ and 5’-TCCACCACCCTGTTGCTGTA-3’. The amount of cDNA in a sample was calculated using the relative standard curve method or ddCT method [9].

Construction of plasmid for immunoprecipitation analysis

The cDNA fragment encoding a FLAG tag was obtained by annealing of the oligonucleotides 5’-atcgactacaaagaccatgacggtgattataaagatcatgacatcgactacaaggatgacgatgacaagtaggggcc-3’ and 5’-cctacttgtcatcgtcatccttgtagtcgatgtcatgatctttataatcaccgtcatggtctttgtagtcgat-3’. The cDNA product was then inserted into the EcoRV and ApaI sites of pSecTagA-sIg-Hepta-Fc [10], yielding pSecTagA-sIg-Hepta-FLAG. To construct ECR-FLAG, a cDNA fragment encoding residues 25–1015 of mouse Ig-Hepta were amplified by PCR from mouse lung cDNA with the following primer set: 5’-atcggtacccacaccaacagagcccattg-3’ and 5’ -atcgatatcatccagaagtatttttaaaag-3’. The PCR product was cut with KpnI and EcoRV, and was then inserted into the same site of pSecTagA-sIg-Hepta-FLAG. To construct Sp-D-Myc, a cDNA fragment encoding full-length Sp-D was amplified by PCR from mouse lung cDNA with the following primers: 5’-atcaagcttatgctgccctttctctccatg-3’ and 5’-atcctcgaggaactcacagataacaaggcg-3’. The PCR product was cut with HindIII and XhoI, and was then inserted into the same site of pCMV-Tag5 (Stratagene, La Jolla, CA, USA). The sequences of all constructs were verified by sequencing.

Cell culture and plasmid transfection

Human embryonic kidney 293T cells were cultured in Dulbecco modified Eagle medium supplemented with 10% fetal bovine serum (FBS), 100 U/ml penicillin and 100 μg/ml streptomycin. Transfection of plasmid vector was performed using TransFectin (Bio-Rad, Hercules, CA, USA) according to the manufacturer’s instructions. To obtain secreted Sp-D-Myc and FLAG-tagged Ig-Hepta deletion proteins, culture medium was changed with Opti-MEM I (Invitrogen, Carlsbad, CA, USA) at 4 h post-transfection and cells were further cultured for 44 h.

Western blot analysis for immunoprecipitates

The protein samples were dissolved in Laemmli buffer (10 mM Tris containing 0.4% SDS, 2% glycerol and 0.02% bromophenol blue, pH 6.8) in the presence of 1% β-mercaptoethanol, heated at 70 °C for 5–10 min, electrophoresed through a 10% SDS-polyacrylamide gel. Proteins were then transferred electrophoretically to Immobilon polyvinylidene difluoride membrane (Millipore) with a semi-dry blotting apparatus (Atto, Tokyo, Japan) using Bjerrum and Schafer-Nielsen buffer (48 mM Tris, 39 mM glycine, 20% methanol and 0.1% SDS) for 45–60 min at ~1.0 mA/cm^2^. The blot was blocked with 5% nonfat dry milk in T-TBS (0.05% Tween 20, 150 mM NaCl and 10 mM Tris-HCl, pH 7.6) for 30 min with agitation at room temperature. After washing 3 times for 10 min each with T-TBS, the blot was treated with the primary antibody in T-TBS overnight at 10 °C. The dilutions of the antibodies were as follows: anti-FLAG M2 monoclonal antibody (1:3,000, Sigma-Aldrich), anti-α-tubulin monoclonal antibody (1:5,000, Sigma-Aldrich), anti–c-Myc monoclonal antibody (9E10; 1:2,000, Roche) and anti-mouse Sp-D rabbit polyclonal antibody (1:3,000, Chemicon, Temecula, CA, USA). After washing 3 times for 10 min each with T-TBS, the blot was incubated for 1 h at room temperature with appropriate secondary antibodies conjugated with horseradish peroxidase (Jackson Immunoresearch, West Grove, PA, USA) diluted 1:15,000–1:30,000 in T-TBS. After washing 3 times for 10 min each with T-TBS, the blot was developed with Luminata Forte Western HRP substrate (Millipore, Bedford, MA, USA) and the signals were visualized using Kodak Image Station 2000R (Eastman Kodak, Rochester, NY, USA). The blots were washed with WB stripping solution (Nacalai Tesque, Kyoto, Japan) for 30 min and were then blocked again with 5% nonfat dry milk in T-TBS for reprobing.

Giemsa staining of cells in bronchoalveolar lavage fluid (BALF).

BALF from both *Ig-Hepta*^+/+^ and *Ig-Hepta*^–/–^ mice were centrifugated at 1000 *g* for 5 min (once for *Ig-Hepta*^+/+^ and 3 times for *Ig-Hepta*^–/–^ mice by adding PBS each time). Then, the supernatant was discarded and pellet was homogenized with fetal bovine serum (FBS) (10 μl for WT and 50 μl for KO mice). About 1 μl of sample was slid using cover glass on the slide glass surface, then air dried. Each sample prepared was then fixed with methanol for 5 min and dried immediately using a dryer. After fixation, the slide glass was added with 1 ml of Wright-Giemsa’s Stain Solution and incubated for 2 min, then added with 1 ml of M/15 Phosphate Buffer Solution, pH 6.4, and incubated for 10–15 min. The liquid was then discarded from the slide glass and washed with water gently for 30–90 seconds, and then dried by using a dryer. Samples were then analyzed under a microscope. Cells were identified using standard morphological criteria.

Counting of cells in bronchoalveolar lavage fluid (BALF).

BALF from both *Ig-Hepta*^+/+^ and *Ig-Hepta*^–/–^ mice were centrifuged at 1000 *g* for 5 min. Supernatant was discarded and pellet was resuspended with PBS. Total cell number counting was conducted using Burke-Turk haemocytometer under a microscope.

**References**

1. Sugaya T, Nishimatsu S, Tanimoto K, Takimoto E, Yamagishi T, et al. (1995) Angiotensin II type 1a receptor-deficient mice with hypotension and hyperreninemia. J Biol Chem 270: 18719-18722.

2. Yagi T, Tokunaga T, Furuta Y, Nada S, Yoshida M, et al. (1993) A novel ES cell line, TT2, with high germline-differentiating potency. Anal Biochem 214: 70-76.

3. Yagi T, Nada S, Watanabe N, Tamemoto H, Kohmura N, et al. (1993) A novel negative selection for homologous recombinants using diphtheria toxin A fragment gene. Anal Biochem 214: 77-86.

4. Goto Y, Sugiyama F, Tanimoto K, Ishida J, Syoji M, et al. (1995) Evaluation of coculture aggregation with TT2 cells for production of germline chimera. Lab Anim Sci 45: 601-603.

5. Atochina EN, Beck JM, Scanlon ST, Preston AM, Beers MF (2001) *Pneumocystis carinii* pneumonia alters expression and distribution of lung collectins SP-A and SP-D. J Lab Clin Med 137: 429-439.

6. Ikegami M, Hull WM, Yoshida M, Wert SE, Whitsett JA (2001) SP-D and GM-CSF regulate surfactant homeostasis via distinct mechanisms. Am J Physiol Lung Cell Mol Physiol 281: L697-703.

7. Ikegami M, Whitsett JA, Chroneos ZC, Ross GF, Reed JA, et al. (2000) IL-4 increases surfactant and regulates metabolism in vivo. Am J Physiol Lung Cell Mol Physiol 278: L75-80.

8. Abe J, Suzuki H, Notoya M, Yamamoto T, Hirose S (1999) Ig-Hepta, a novel member of the G protein-coupled hepta-helical receptor (GPCR) family that has immunoglobulin-like repeats in a long N-terminal extracellular domain and defines a new subfamily of GPCRs. J Biol Chem 274: 19957-19964.

9. Livak KJ, Schmittgen TD (2001) Analysis of relative gene expression data using real-time quantitative PCR and the 2^-DD^*^C^*^T^ method. Methods 25: 402-408.

10. Abe J, Fukuzawa T, Hirose S (2002) Cleavage of Ig-Hepta at a "SEA" module and at a conserved G protein-coupled receptor proteolytic site. J Biol Chem 277: 23391-23398.
